# Recent Advances of Graphene-Derived Nanocomposites in Water-Based Drilling Fluids

**DOI:** 10.3390/nano10102004

**Published:** 2020-10-11

**Authors:** Rabia Ikram, Badrul Mohamed Jan, Jana Vejpravova, M. Iqbal Choudhary, Zaira Zaman Chowdhury

**Affiliations:** 1 Department of Chemical Engineering, University of Malaya, Kuala Lumpur 50603, Malaysia; badrules@um.edu.my; 2 Department of Condensed Matter Physics, Faculty of Mathematics and Physics, Charles University, Ke Karlovu 5, 121 16 Prague 2, Czech Republic; jana@mag.mff.cuni.cz; 3 HEJ, Research Institute of Chemistry, International Center for Chemical and Biological Sciences, University of Karachi, Karachi 75270, Pakistan; iqbal.choudhary@iccs.edu; 4 Panjwani Center for Molecular Medicine and Drug Research, International Center for Chemical and Biological Sciences, University of Karachi, Karachi 75270, Pakistan; 5 Nanotechnology & Catalysis Research Centre, Deputy Vice Chancellor (Research & Innovation) Office, University of Malaya, Kuala Lumpur 50603, Malaysia; dr.zaira.chowdhury@um.edu.my

**Keywords:** nanotechnology, graphene-derived materials, mud cake, rheology, effect of nanocomposites, fluid loss, water-based drilling fluids

## Abstract

Nanocomposite materials have distinctive potential for various types of captivating usage in drilling fluids as a well-designed solution for the petroleum industry. Owing to the improvement of drilling fluids, it is of great importance to fabricate unique nanocomposites and advance their functionalities for amplification in base fluids. There is a rising interest in assembling nanocomposites for the progress of rheological and filtration properties. A series of drilling fluid formulations have been reported for graphene-derived nanocomposites as additives. Over the years, the emergence of these graphene-derived nanocomposites has been employed as a paradigm to formulate water-based drilling fluids (WBDF). Herein, we provide an overview of nanocomposites evolution as engineered materials for enhanced rheological attributes in drilling operations. We also demonstrate the state-of-the-art potential graphene-derived nanocomposites for enriched rheology and other significant properties in WBDF. This review could conceivably deliver the inspiration and pathways to produce novel fabrication of nanocomposites and the production of other graphenaceous materials grafted nanocomposites for the variety of drilling fluids.

## 1. Introduction

Over the years, the influential production of nanomaterials through nanotechnology has prominently contributed to the advancement of expertise in many industries. Numerous studies have been performed to address the impact of significant applications of nanomaterials in drilling fluids [1]. Lately, materials sciences and engineering accomplished remarkable progress in the field of nanocomposites fabrication with enriched physical, chemical, and mechanical properties [2]. A widespread range of studies were directed at processing these nanocomposites [3]. The combination of graphene in nanocomposites has resulted in their enrichment of mechanical strength, thermal stability, electrochemical activity, electrical conductivity as well as gas barrier properties [4,5]. Manifestation of graphene in these composites has established unique properties via increasing functional groups on the material’s surface for a variety of drilling fluids [6]. A smooth process of drilling requires an appropriate well control system, suitable usage of a blowout preventer, and proper formulation of drilling fluids [7]. To motivate drilling of wellbore, drilling fluids are utilized to circulate in the borehole to ensure an efficient drilling process [8]. Typically, three substantial types of drilling fluids or drilling muds have been reported: water-based drilling fluids (WBDF), oil-based drilling fluids (OBDF), and synthetic-based drilling fluids (SBDF) [9,10].

These drilling fluids motivate the drilling process by transporting suspended cuttings back to the surface, cooling the drill bit and providing stabilization for the rock formation, and also controlling the pressure inside the well [11]. In addition, drilling fluids are reported to prevent corrosion of the equipment and mud cake formation on the wellbore wall as well preserving the cuttings to settle down if the circulation stops abruptly [12]. Due to the inefficient methods of well cleaning, the cuttings inclined to deposit when circulation stops causes the bit to become incapable of operating properly and the drills fresh formation becomes buried under those deposited cuttings, ultimately leading to a delay in the drilling process [13]. Conjointly, drilling fluids are required to remove heat from the bit and transfer the heat to the surface to cool the drill bit [14]. In the case of improper handling, the bits performance is diminished and eventually become damaged [15].

One of the major advantages of nanocomposites include affirmative changes by adding even a small amount of graphene filler in the presence of miscellaneous polymeric matrices [16]. Therefore, processing of nanocomposites is critical for environmental and cost-friendly rheological behavior. The most promising features, such as plastic viscosity, yield point, shear rates, and gel strength along with filtrate loss and mud cake thickness, are frequently assessed for the rheological performance of drilling fluids [17,18]. This review highlights the importance of graphene-derived nanocomposites with polymer, active carbon, metal, metal oxide, carbon fiber, and their applications in WBDF. A summarized effect of nanocomposites is abridged that incorporates nano-sized particles into a matrix of a standard material for drilling fluids. Based on the functionality of these nanocomposites, proper treatment of drilling fluids rheology is indeed imperative, so they tend to deliver their functions for a smooth drilling process.

## 2. Role of Significant Nanocomposites in Drilling Fluids

There have been advances in fabricating nanocomposites composed of a multiphase solid materials, where one of the dispersed phases is in the nanometer-scale dimension [19] and the other is a major phase, such as ceramic, metals, or polymers [20]; carbon-carbon composites [21] and nanocarbon [22] are combined as a matrix material.

For the past years, several studies have been conducted on the applications of numerous nanocomposites in drilling fluid such as polyacrylamide/clay nanocomposite [23], nanocarboxymethyl cellulose/polystyrene core-shell nanocomposite [24], polymer nanocomposite [25], nanosilica polymer composite [17], TiO_2_-polyacrylamide [26], clay nanocomposite [27], ZnO-clay composite, and ZnO-Am nanocomposite [27,28]. The outcome of these studies presented a homogenous dispersion of nanocomposites in the drilling fluids to serve multiple functions simultaneously, for instance, fluid loss control, high thermal stability, enhanced rheological performance and a reduction in mud cake thickness. Furthermore, common methods, such as injection molding, solvent processing, chemical and vapor techniques, in situ polymerization, melt blending, template synthesis, spray pyrolysis, and sol-gel methods have been utilized to improve nanophase dispersion while processing graphene-derived nanocomposites [2,3,6,16]. The application of nanocomposites as additives, such as stabilizers, surfactants or ionic liquids, have had a productive effect on the rheological properties as compared to drilling fluids with single nanoparticles [5,28].

### 2.1. Effect of Silica Nanocomposites

Cheraghian et al. [29] summarized their research on Clay/SiO_2_ nanocomposite as compared to SiO_2_ nanoparticles to determine the rheological properties of drilling fluids. Their work fabricated Clay/SiO_2_ nanocomposite consisting of nano-fumed silica and sodium bentonite synthesized through an effective hydrothermal method. The effects of SiO_2_ nanocomposites in rheological tests at low and high temperatures showed that properties, such as apparent viscosity, plastic viscosity, yield point, and gel strength, increase as the concentration of nanocomposite is augmented. The measured values were higher than the drilling fluid with SiO_2_ nanoparticles and base fluid. Due to the small size of the SiO_2_ nanocomposites, the plugged pores were more effective than the SiO_2_ nanoparticles which then formed a thin and impermeable mud cake to reduce filtrate loss control. Therefore, the SiO_2_ nanocomposites also showed remarkable filtration control at high temperatures. By the additivation of a minute amount of SiO_2_ nanocomposites (0.1 wt.%), a sufficient and lower fluid loss control at a recommended value of 15 mL by the American Petroleum Institute (API) was displayed [30]. Consequently, it was established that SiO_2_ nanocomposites are efficient at high pressure–high temperature (HPHT) wellbore conditions of drilling fluids.

### 2.2. Effect of Copper Oxide Nanocomposites

**Figure 1:**
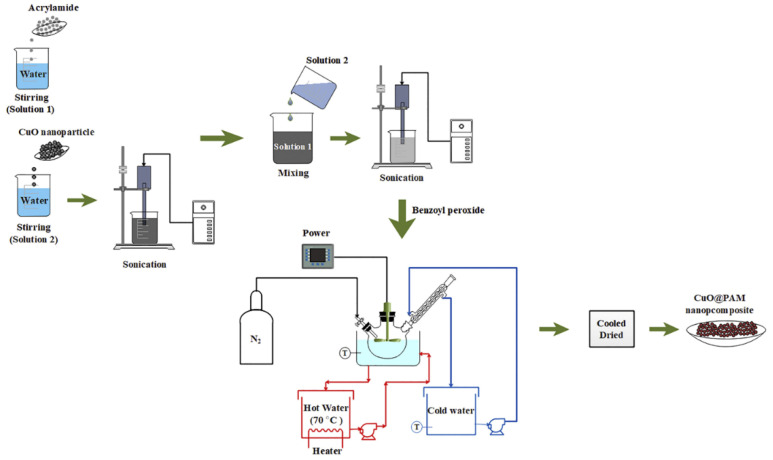
Synthesis of copper oxide/polyacrylamide (CuO/PAM) nanocomposite through the solution polymerization process. Reprinted with permission from [31]. Copyright Elsevier, 2019.

Several studies have been implemented on the application of copper oxide nanocomposites in drilling fluids with better results in water-based muds [23,26]. Saboori et al. [31] studied the influence of copper oxide/polyacrylamide (CuO/PAM) nanocomposite synthesized through a solution polymerization method as shown in Figure 1. The improvement in rheology was observed together with the thermal conductivity of bentonite drilling fluids. The outcome of this study represented high viscosity by increasing the concentration of CuO/PAM nanocomposite due to the usage of PAM, which is known as a viscosifier in drilling fluids. Meanwhile, an increase in CuO/PAM nanocomposite concentration has been resulted in reduction of fluid loss volume and mud cake thickness due to the appropriate pore sealing by nanocomposites with an average size of 55.4 nm. Therefore, the application of CuO/PAM nanocomposite has enhanced the thermal conductivity due to the heat transfer between the drill bit and the drilling fluid. It has been proved that instantaneous function of CuO/PAM nanocomposite was achieved as an additive in the drilling fluids [32,33].

### 2.3. Role of Titanium Dioxide Nanocomposites

Titanium dioxide (TiO_2_) as metal oxide nanoparticles have been evidenced as chemically and physically stable. Also, they are exposed as nontoxic by providing high thermal conductivities [34]. Based on the unique characteristics of TiO_2_, Sadeghalvaad et al. [26] produced TiO_2_/polyacrylamide (PAM) nanocomposites through the solution polymerization method in order to increase the properties of nanocomposites which acted as viscosifier and thermal conductivity enhancers. The addition of this nanocomposite is exhibited as a nanofluid that stayed in liquid form, regardless of ambient temperature as opposed to changing into solid form in drilling fluid [35]. This property was observed for the reason that capability of TiO_2_ nanoparticles in transferring heat efficiently occurred due to the Brownian motion [36]. Besides, TiO_2_ nanocomposites also contributed to enhance viscosity and reduced the amount of fluid loss and mud cake thickness. By increasing TiO_2_ nanocomposites concentration, the rheological properties of drilling fluids were modified excessively [26].

### 2.4. Effect of Grass and Other Additives to Improve Rheological Properties

Drilling wastes are considered as the second largest volume of waste generated in the oil and gas industry [37]. Two major generated wastes include drilling cuttings and drilling fluids. These wastes are required pre-treatment before being disposed of in order to protect humans and the environment. Correspondingly, researchers developed an idea of producing an eco-friendly drilling fluid system which entails the same striking rheological and filtration properties among drilling fluids without non-toxic chemical additives [38].

In recent times, researchers have inspected the application of powdered grass in drilling fluids for refining the rheological and filtration performance. However, some studies are limited to the application at low pressure-low temperature (LPLT) conditions. Investigation at HPHT conditions was declared to be important in addressing the ability of this eco-friendly drilling fluid as compared to bottom hole conditions which were found likely to be at HPHT. Therefore, the application of different powdered grass concentrations in drilling fluids under LPLT and HPHT conditions were conducted by Al-Hameedi et al. [39]. Effective concentrations of 0.5%, 1.0%, and 1.5% of powdered grass were added separately to the base fluid which only consisted of bentonite, NaOH, and water. It showed that the addition of powdered grass in each concentration increased the plastic viscosity, yield point, and gel strength of the drilling fluids as compared to the base fluid. In addition, the powdered grass drilling fluid also showed impressive filtration properties at LPLT and HPHT conditions. During LPLT condition, 0.5% of powdered grass showed better fluid loss control and equated to 1.0% and 1.5% of powdered grass. Whereas at HPHT conditions, a 43% reduction in fluid loss volume was recorded by using a 1.5% concentration of powdered grass as compared to the base fluid. Furthermore, an impermeable and thin mud cake was formed in both conditions. Hence, powdered grass additive has revealed great potential for use as a fluid loss control agent.

### 2.5. Applications of Various Nanocomposites in Drilling Fluids

The depletion of hydrocarbons in conventional shale has caused a surge in investigation of reservoirs that exhibited severe conditions such as HPHT, high salinity [40], and widely distributed nano-sized pores. Numerous mathematical models such as Bingham plastic, Newtonian fluid, and Herschel-Bulkley fluid have been reported as reliable sources to evaluate hydraulic parameters of drilling fluids [41]. This exploration driven to meet the growing demand for energy consumption by consumers.

In order to retrieve oil beneath a layer of earth, drilling should be completed through a successful drilling process. However, drilling through conventional drilling fluids has resulted in several problems, such as excessive fluid loss [42], thick mud cake [43], and poor rheological properties at HPHT, which has led to the intervention of nanomaterials for transformation of conventional drilling fluids. By captivating into the progress of nanotechnology, nanocomposites application has improved rheological and filtration properties in drilling fluids [25,44]. Several types of nanocomposites as additives have helped to increase wellbore stability, forming impermeable mud cake [45], reduced fluid loss into formation [46], and enhanced the viscosity of the drilling fluids [31]. Though nanoparticles were examined due to the fact of their contribution in remodeling the rheological properties by plugging nano-sized pores which helped to control the fluid loss [47]. Numerous studies have indicated that the application of nanocomposites produced better drilling fluid properties. A summary of recent studies of drilling fluids is abridged along with several nanocomposites as presented in Table 1.

## 3. Variation of Rheological Properties by Nanocomposites

Combinations of nano-sized particles are triggered by joining with other solid particles present in the mud system, such as bentonite, either directly or via intermediate chemical linkages [59]. Since nanomaterials have revealed a high surface area which greatly enhances interaction between nanocomposites and the matrix of the mud system [60], recent studies have shown high concentrations of nanocomposites and their effects at different temperatures in WBDF [61]. By increasing the number of nanomaterials in drilling fluids, sufficient hole cleaning has improved which substantially reduces many drilling problems. Subsequently, adequate amounts of nanocomposites were evaluated as an important factor to ensure drilling operations efficiently and without adding extra pressure on the drilling pump [62]. Also, the existence of nanocomposites in the drilling mud system has produced a strong repulsion force among negatively charged bentonite particles to improve the rheology of drilling fluids. Correspondingly, the increase in strong repulsion prevented agglomeration. Hence, a strong clay platelet network was formed [63].

### 3.1. Gel Strength

**Figure 2:**
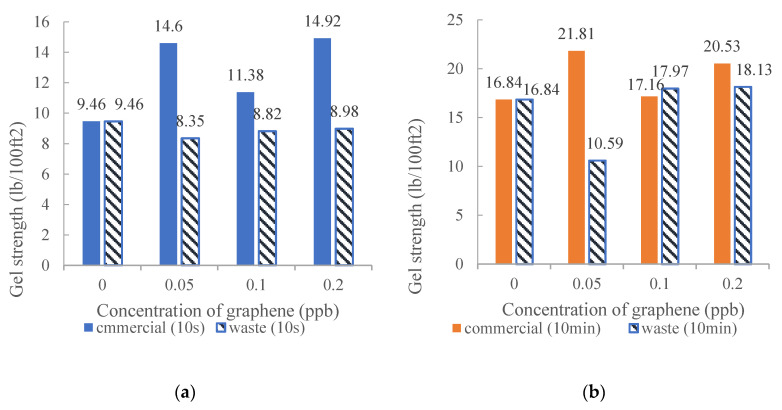
Gel strengths of different concentration of graphene before aging: (**a**) 10 s and (**b**) 10 min. Reprinted with permission from [61]. Copyright IOP Publishing, 2020.

**Figure 3:**
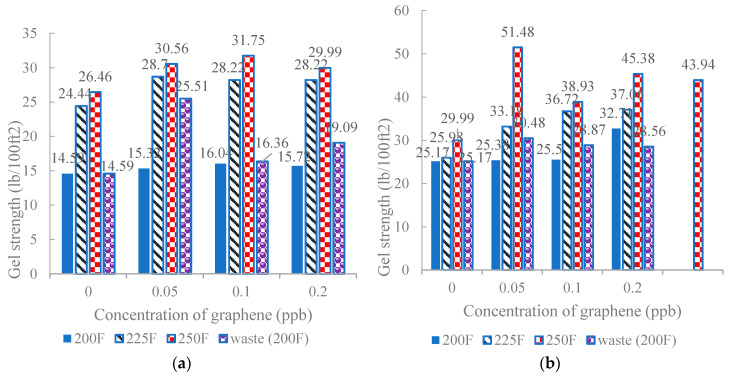
Gel strengths of different concentrations of graphene after aging: (**a**) 10 s and (**b**) 10 min. Reprinted with permission from [61]. Copyright IOP Publishing, 2020.

Gel strength allows the drill cuttings to suspend as soon as the circulation of the mud stops unexpectedly [64]. It prevented a critical mechanism of the wellbore to collapse. By the addition of nanocomposites in the drilling fluid, it facilitated the drilling fluid system to create a gel structure quicker than the base fluid. Moreover, studies have displayed an increase in gel strength by increasing the concentration of nanocomposites in WBDF as disclosed in Figure 2 and Figure 3 [61].

### 3.2. Filtrate Loss

**Figure 4:**
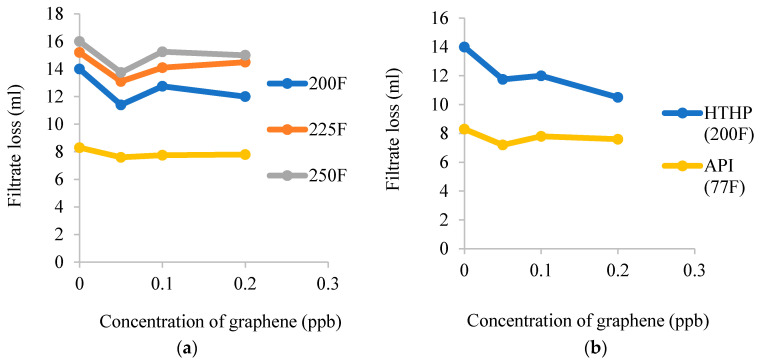
High pressure-high temperature (HPHT) filtrate loss at different concentrations of (**a**) commercial graphene and (**b**) waste graphene. Reprinted with permission from [61]. Copyright IOP Publishing, 2020.

Filtrate loss of drilling fluids was measured under LPLT or HPHT using a filter press following the procedure stated in the recommending practice API 13B-1 [65]. Nanocomposites lower than 100 nm in size were established to mix with nanoporous medium and were found challenging to be tailored into conventional drilling fluids performance [66]. Later, by increasing the concentration of nanocomposites, a thin, stiff, and impermeable mud cake was formed. Resultantly, the filtrate loss was greatly reduced into the rock formation. Barry et al. [67] revealed a decrease in filtrate loss volume which contributed by intensification of electrostatic forces between negative ions of nanocomposites to avoid flocculation with other particles as evidenced in Figure 4. Hereafter, it produced a thin and impermeable mud cake on the surface of the wall.

### 3.3. Shear Rate

Since rheology describes the deformation of an ideal fluid under the influence of stress, it is equally important to know the shear rates of drilling fluids to understand their rheological performance. Researchers have examined carbonaceous materials effects for improved rheology of WBDF. For example, carbon nanotubes were considered to increase the shear stress which found to be proportional to the shearing rate. It was due to the better dispersion of base-fluid mud combined with carbon nanotubes at high shear rates [68].

Similarly, the yield stress and viscosity were up surged due to the resistant of fluid structure malformation as compared to the conventional drilling muds. Also, studies have reported low shear rate viscosity profiles of WBDF by adding nanocomposites. For instance, Vallejo et al. [69,70] conducted studies of loaded dispersions of carbon black (0.25%), nano-diamonds (0.50%), graphite/diamond (1%), and graphene nanoplates (1.5%) to analyze shear rates of 24 nanofluids. By increasing temperature, the samples presented a decrease in viscosity of 76% to 84% at a shear rate of 57.4 s^−1^ and 79% to 83% at a shear rate of 489 s^−1^. 

Likewise, proportional carbon-based nanofluids presented a decline of dynamic viscosity at 70 K for 84% and a shear rate increase of 57.4 s^−1^, hence, they examined the shear rates between 10 to 100 s^−1^ for elevated viscid conditions [71]. In addition, Sayindla et al. [72] evaluated improved rheological properties in field conditions such as shear rates below 400 s^−1^ for WBDF viscosity profiles. The encountered low shear rate viscosity profile was observed below 400 s^−1^ along with rheological performance comparison between WBDF and OBDF.

**Figure 5:**
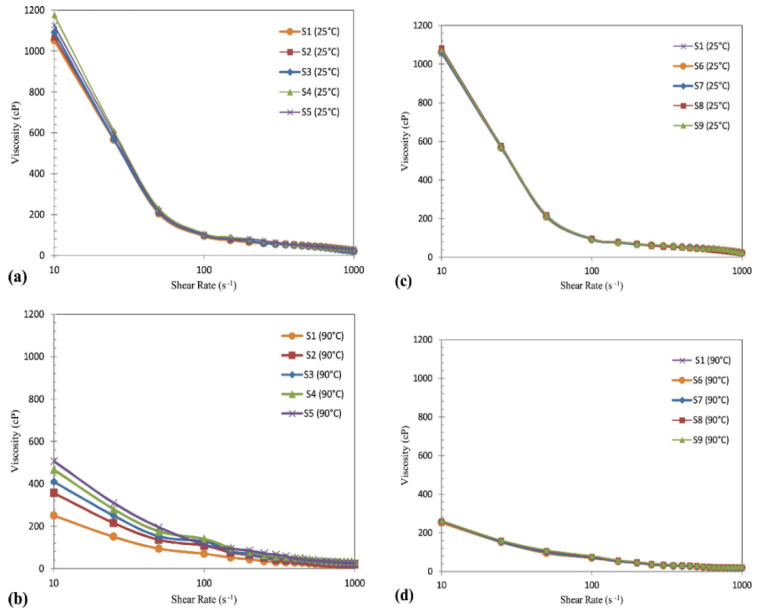
Temperature effects on viscosity: (**a**,**b**) nano clay/SiO_2_ WBDF (S2-S5) and (**c**,**d**) SiO_2_ WBDF (S6-S9) at 25 and 90 °C compared to base fluid (S1). Reprinted with permission from [29]. Copyright Elsevier, 2018.

Studies have inspected the temperature effect on viscosity of nanoclay/SiO_2_ water-based muds (S2-S5), their comparison with base mud (S1) and SiO_2_ water-based muds (S6-S9) at 25 and 90 °C. It was detected that nanoparticles enhanced the viscosity of the muds. Conversely, viscosity of nano clay/SiO_2_ water-based fluids was decreased with accelerating shear rate. The viscosity of 1000 cP at shear rate of 10 s^−1^ was observed at 25 °C, while viscosity decreased at 90 °C to 200 cP for base fluid (S1). Moreover, clay/SiO_2_ water-based mud presented the viscosity of 1200 cP at 25 °C, though it dropped to 600 cP at a higher temperature of 90 °C. By increasing the concentration of these nanomaterials in WBDF, observed viscosity was enhanced at both temperatures which can be seen in Figure 5 [29].

## 4. Graphene Impacts in Drilling Operations

Graphene as a unique material with a one atom thick sheet of sp^2^ hybridized carbon atoms is well known due to the fact of its remarkable properties such as physical, electrical, optical, electrochemical, large surface to volume area, thermal stability, and high mechanical strength. These properties make graphene a unique material for a wide range of industrial applications [73]. For example, high electrical conductivity and electrocatalytic efficiency have made graphene an exceptional tool for electrochemical applications. Likewise, other properties, such as high hydrophobicity, prominent electric conductivity, and mechanical strength, have testified to graphene’s incorporation as a sensing element for biosensors. Despite these wonderful properties, synthesis of graphene sheets is difficult to produce at a large scale. Due to the poor dispersion of graphene in organic solvents [74], graphene oxide (GO) and reduced graphene oxide (rGO) have been found way more favorable as compared to graphene in drilling fluids.

**Figure 6:**
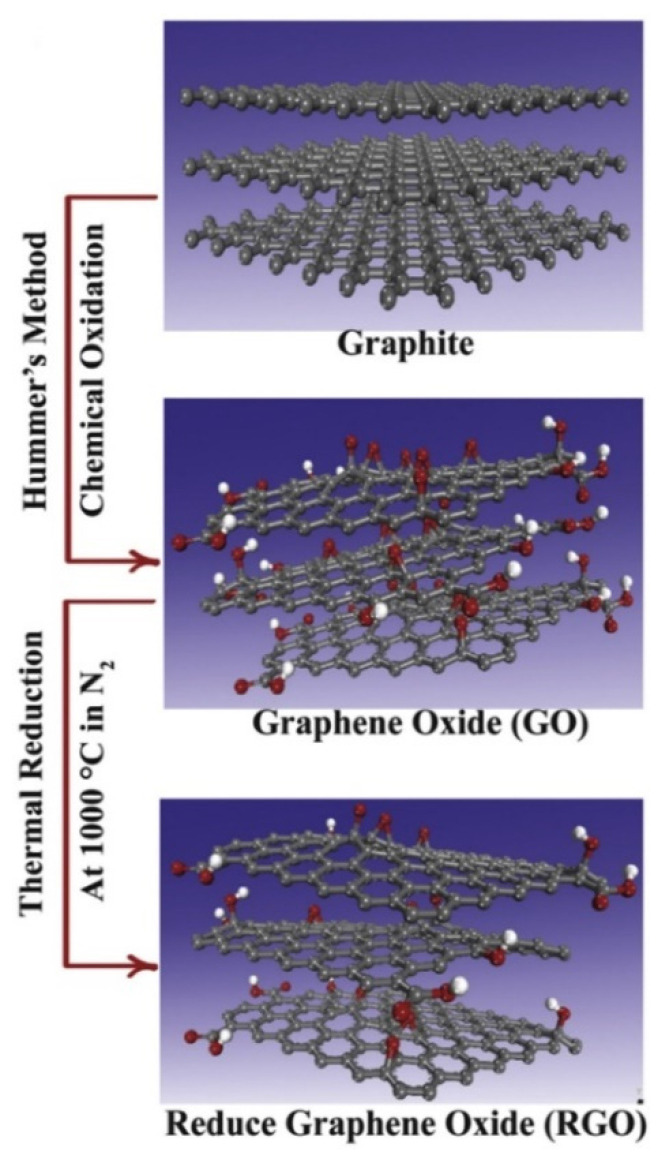
Schematic formation from graphite, GO to rGO. Reprinted with permission from [78]. Copyright Elsevier, 2019.

Earlier studies have been produced GO either by modified Hummers method through oxidation of graphite by using sulfuric acid, nitric acid, and potassium manganate [75] or by dispersion of GO precursors in water and aqueous KOH solution [76]. Li et al. [77] proposed a method without any addition of polymeric or surfactant stabilizers to ensure notable dispersion of graphene and concluded a stress-free way to produce aqueous graphene dispersion for large-scale production. A significant role of graphene-derived structures is represented in Figure 6.

The presence of oxygenated groups in a GO structure was produced with higher solubility and well-dispersed GO nanosheets in water and organic solvents [79]. Simultaneously, these oxygenated groups distressed the electrical, mechanical, and electrochemical properties of GO which made it slightly different from graphene [73].

Likewise, rGO was produced through thermal and electrochemical reduction of GO [80]. William et al. [81] synthesized rGO through photocatalyzed reactions using TiO_2_ as a catalyst stimulated with UV light. This conversion of rGO was testified to minimize the number of oxygenated groups presented in the structure of GO to gain attributes relatively similar to graphene [82]. As compared to the extraordinary properties of graphene, GO and rGO have affirmed advantageous roles to vital industries. Keeping up to date with current technology advancements, novel perspectives have been invented in favor of enhancing the quality of graphene-derived materials [83]. These approaches have been involved the combination of composite materials to counterbalance merging of conventional ceramics, metal alloys, and many polymeric materials. On the contrary, grouping of two or more materials is found to be a superlative classification of composites in the form of fibers, sheets or particles for their fabrication into the matrix phase [84]. While for nanocomposites, one of the materials is composed of dimension less than 100 nm [19].

Although graphene has attracted substantial attention due to the fact of its distinctive properties such as extraordinary mobility and conductivity, contrarily, impurities have been found in order to recover its functionality and electrochemical activity [85]. Therefore, graphene-based nanocomposites including inorganic nanostructures, conducting polymers, and organic materials have been combined for enriched mechanical strength, electrical conductivity, and thermal stability [86]. Lawal et al. [78] stated that a small quantity of graphene filler was needed to incorporate into the polymer matrix in order to enhance the properties and characteristics of nanocomposites. While addressing the drilling operations in extreme conditions, suitable additives are required to use in WBDF to avoid decomposition and better performance of the drilling fluids during drilling process. Nevertheless, due to the current advances in technology, novel nanocomposites have been selected to utilize as additives for modification of rheological properties as well as the filtration behavior of drilling fluids [87].

In 2004, extraction of graphene was successfully done by Andre K. Geim and Konstantin Novoselov which later won them the Noble Prize in 2010 [88]. The extraction method involved peeling off graphite flakes from substances with the least defects [89]. Graphene is considered as a wonderful material due to the fact of its unique attributes and thickness of an atom with high surface-area-to-volume ratio [90] which has made it suitable for many oil and gas merged industrial applications. Many studies have been conducted on refining the rheological and filtration properties of drilling fluids through incredible deployment of graphene-grafted nanoparticles [23]. The application of graphene and its derivatives was reported for rheological performance augmentation and stability of the shale [11]. Due to the fact of its nano-sized particles, it is a favorable fluid loss control additive in contrast to bentonite that is usually offered in base fluids [91]. These nano-sized particles are accomplished to plug the nano-sized pores that are present in the fluid. As a result, enriched mud cake with thin and low permeability characteristics is formed. However, some studies have exposed that graphene tends to flocculate and cause poor dispersion of nanoparticles in the drilling fluid system. This problem is resolved through the application of graphene derivative such as GO and its dispersion in an aqueous solution due to the fact of its highly hydrophilic possessions [92]. Aftab et al. [28] categorized rheological properties using graphene nanoparticles for noteworthy filtrate volume which was remained stable at HPHT conditions as compared to the base fluid. Since graphene contained a high-surface-to-volume ratio, considering a small amount of graphene was found to be sufficient enough to increase the thermal conductivity, heat tolerability, and the effectiveness of interaction among rock surfaces [89]. Hence, the drill bit was able to transfer the heat generated during the drilling process to cool off the bit. Consistently, in a research by Friedheim et al. [93], GO was proved as a viable shale inhibitor option at HPHT conditions due to the fact of its ability to mitigate the swelling effect in shale formation It was triggered by the interaction of water between clay minerals which existed in the shale and enhanced wellbore stability to prevent from collapse.

### 4.1. Graphene-Derived Nanocomposites in WBDF

**Figure 7:**
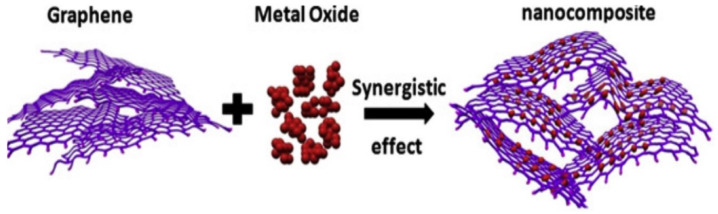
Fabrication of graphene-derived nanocomposite from metal oxide. Reprinted with permission from [78]. Copyright Elsevier, 2019.

The combination of graphene with composites have been utilized to develop superlative properties as a filling agent to advance the applications of nanocomposites in WBDF. A symbolic role of graphene-derived nanocomposites was tested to minimize fluid loss and notable effects on lubricity, viscosity, yield stress, shear rate, etc. Efforts of graphene flakes dispersion in WBDF resulted as unideal remediation for drilling fluids. In contrast, dispersion of graphene-derived nanocomposites compacted the interlocking of diverse materials to maintain desired pore-plugging through mud cake formation. It allows nanocomposites to serve multiple rheological functions with minute quantities due to the fact of their well-exfoliation and enriched functional characteristics in any system as presented in Figure 7 [35].

A summary of studies has displayed the rheology of drilling fluids by utilizing nanocomposites as filtration reducing agent [24], heat resistant [34], viscosifier [42], shale inhibitor [51], weighing additives through the magnetic field into an environmental responsive product [56], nano-emulsion lubricant for strong inhibition [23], and desulfurizing agent to remove H_2_S from drilling fluids due to the fact of their high porosity and surface area [94]. The fabrication of graphene nanocomposites with polymers, organic, inorganic, and carbon materials as well as summarization of their progress for rheological and filtration properties of WBDF is displayed in Table 2.

#### 4.1.1. Graphene-Polymer Nanocomposites

**Figure 8:**
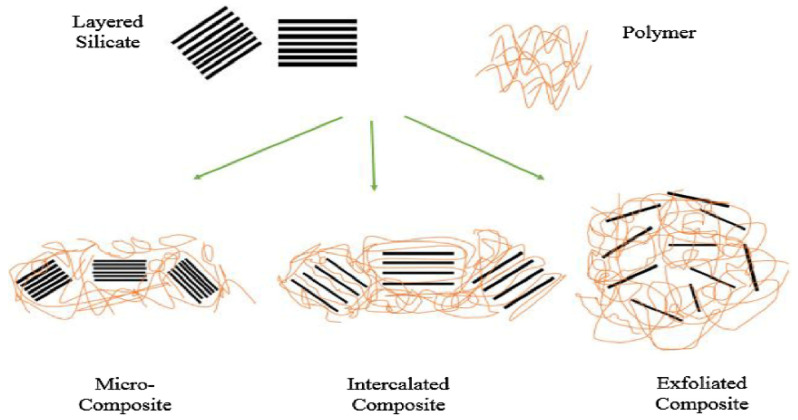
Assembling of polymer–clay nanocomposites through in situ intercalation, melt intercalation, and exfoliation techniques. Reprinted with permission from [96]. Copyright Elsevier, 2018.

Various studies for graphene–polymer nanocomposites have been developed by chemical or electrochemical polymerization of the monomers in the presence of graphene. Considerable polymer nanocomposites research has motivated on uncovering synthesis routes, for instance, in situ polymerization, solvent blending, melt compounding to fabricate graphene-based materials. Quantitative dispersion of these materials determines the structural deformation to stabilize their properties and unnecessary functionalization into polymer matrix. Concerning this, untangling of sheets during polymer dispersion into other materials incapacitates their unique properties [95]. Furthermore, ultrasonication has deployed better graphene dispersion in the polymer matrix as a nanofiller. On the other hand, consideration was taken for size and wt.% of desired nanomaterial. Individual types of polymer–clay nanocomposites and their interaction among the fillers are demonstrated in Figure 8 [96]. Several examples of graphene–polymer nanocomposite in WBDF are enumerated in Table 2.

#### 4.1.2. Graphene-Activated Carbon Nanocomposites

Fabrication of graphene on activated carbon is employed for commercial usage due to the low cost and availability of activated carbon [97]. Besides, activated carbon has intensified performance due to the fact of its pore structure and large surface area. The resulting nanocomposite presented high performance, suitable yield point, plastic velocity as well as thin mud cake formation [98]. Examples of graphene-activated carbon nanocomposites as a high enactment material in WBDF are listed in Table 2.

#### 4.1.3. Graphene-Metal Nanocomposites

The incorporation of metals as composite, such as copper, gold, and iron, into graphene are considered as the next generation conductors due to the fact of their room temperature tolerance and high resistivity as compared to conventional metals. The effect of graphene-metal nanocomposites has upgraded rheological properties using different concentrations of particles sizes less than 50 nm [99]. A great number of functional groups have been incorporated due to the metal nanocomposites which resulted in low shear thinning and a decrease in mud filtrate. Due to the fact of their high thermal conductivities that dissolved heat efficiently, they have added benefits for upraised electrochemical properties and analytical performances in drilling operations [100]. Influence of these graphene-metal nanocomposites for WBDF are briefly tabulated in Table 2.

#### 4.1.4. Graphene-Metal Oxide Nanocomposites

Lately, graphene-metal oxide nanocomposites have been widely used as an alternative with cost-friendly results in drilling fluid applications [101]. Due to the combination of graphene into pores of metal oxygenated groups, these nanocomposites exhibited a great tendency to tolerate HPHT conditions of drilling operations. Recent studies have contributed to an enhanced usage of metal oxides for the fabrication of graphene nanocomposite for testified high-energy density. Rheological behavior of these nanocomposites has immensely influenced WBDF [102,103]. A significant role of graphene-metal oxide nanocomposites for value-added properties of WBDF is given in Table 2.

#### 4.1.5. Graphene-Fiber Nanocomposites

**Figure 9:**
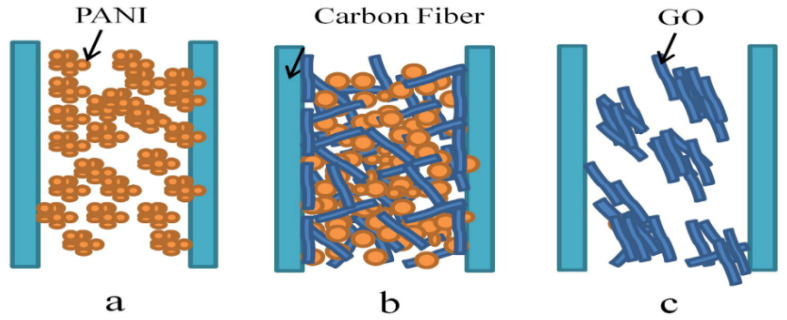
Representative dispersion of matrix: (**a**) random distribution of polyaniline (PANI), (**b**) enhanced dispersion of hybrid fillers due to the electrostatic interaction, (**c**) poor dispersion of GO due to the large particles agglomeration. Reprinted with permission from [105]. Copyright Elsevier, 2016.

Graphene–fiber nanocomposites are formed through direct covalent bonding of carbon fiber with graphene to advance WBDF as compared to bentonite-formulated base fluids. Studies have uncovered a great effect of these nanocomposites by novel incorporated fibers for minimizing fluid loss, reduced mud cake damage, and enhanced performance of drilling fluids. Potential studies were presented for sealing of wellbores and fluid production to prevent leakage to the surface resulting in low costs, low environmental risks, persistent wellbore reliability, and well cementing modern technologies [104]. In addition, hybridized nanocomposites enhance the thermal stability of conventional drilling fluids which shows a reduction in mud filtrate and modification of nano-additives in drilling fluids. An example of polyaniline (PANI)-GO nanocomposite dispersion to avoid self-aggregation is presented in Figure 9 [105,106].

Several researchers have investigated the role of graphene-fiber nanocomposites in WBDF which are briefly entailed in Table 2.

**Table 2:** Summary of the graphene nanocomposites in WBDF.

Graphene-Derived Nanocomposites	Synthesis Routes	Conditions & Outcomes	References
Graphene-polypropylene (PP) nanocomposite	Melt mixing	Enhanced PV versus SR, 20~5000 s^−1^ for nanocomposites at 200 °C. The PV of PP was observed 289 Pa s at 300 s^−1^ SR, which increased up to 513 Pa s due to the stronger interaction of the PP matrix with GO nanocomposite.	[107]
Graphene-acrylonitrile butadiene styrene resin (ABC) nanocomposite	Facile coagulation method	An increase of PV and mechanical modulus was observed due to the graphene nanocomposite.	[108]
Graphene-polyester nanocomposite	Partial pyrolysis	An enriched RP was observed due to the nanocomposite as compared to graphite.	[109]
Graphene-polyurethane nanocomposite	Solution mixing method	0.5–3 wt.% qualitative expansion was presented in the frequency of RP.	[110]
Graphene-low density polyethylene nanocomposite	Melt extrusion and film casting	Established PV, ST, viscoelasticity at 140 ℃.	[111]
GO-Fe_2_O_3_/Al_2_O_3_ nanocomposite	Vertical bed method	Nanocomposite reduced the FL from 20 mLto 15 mL and MCT from 0.3 mm to 0.1 mm of WBDF with 0.02%.	[112]
GO-ZrO nanocomposite	Microwave synthesis	Enriched HPHT applications using a high-temperature range of 330 °F.	[113]
rGO-SnO_2_ nanocomposite	Ultrasonic synthesis	Improved RPs were reported with the effect of vol% of rGO–SnO_2_ nanocomposite (three different ratios: 1:7, 1:8, 1:10) in base fluid for PV, ST, ranging from 0 to 10,000 s^−1^ at 25 °C.	[114]
GO-ZnO nanocomposite	Chemical synthesis	A desirable increase of PV (5–28%), YP (25–42%), GS (25–33%), and a considerably reduced FL were examined.	[66]
rGO-thermally polypyrrole nanocomposite	In situ polymerization	RP of rGO-thermally polypyrrole nanocomposite was determined using a cone-plate method with ratios (100:1, 100:3, and 100:5%) and temperature (25–180 °C), and represented an increase of ST and PV due to the addition of thermally reduced GO sheets into polypyrrole.	[115]
GO-polyacrylamide (PAM) nanocomposite	Chemical synthesis (polymerization)	Nanocomposite influenced FL at LPLT and HPHT which was reduced up to 38.96% and 34.36, respectively. A noteworthy decrease in FL, MCT treated with 1.5 wt.% nanocomposite.	[116]
GO-hydrolyzed polyacrylamide nanocomposite	Chemical synthesis	Addition of GO increased PV, the effect was notable at elevated temperatures. Addition of 0.1 wt.% of GO enhanced PV by 47% and 36%, respectively, at 85 °C and 25 °C. GO increased the thermal stability due to the electrostatic hydrogen bonding among nanocomposite functional groups. After ageing for 30 days at 80 °C, PV of the composite’s solution decreased very slightly, while a 59% reduction was observed for pure polymer solution.	[117]
GO nanocomposite	Chemical synthesis	Reduced FL was observed using low concentration of GO nanocomposite.	[118]
**Applications of Other Graphenaceous Materials in WBDF**			
GO/polyanionic cellulose polymers	Hummers method	FL of 6.1 mL over 30 min, MCT ~20µm/FL of 7.2 mL, MCT ~280 µm, high-temperature stability with better-quality RP.	[16]
GO	Hummers method	Concentration of GO increased from 0.2 wt.% to 0.6 wt.%, PV of GO aqueous dispersion noticeably increased, whereas there was no obvious change of YP and GS.	[119]
GNP	Hydrothermal technique	Amended RP was presented at HPHT due to the low friction between nanoplates.	[28]
Graphene/MgO/TiO_2_	Hydrothermal technique	An increase of GS (92%) and PV (253%) by adding MgO (2%) and graphene (75%) was observed.	[120]
Graphite–Al_2_O_3_	-	Upgraded drilling mud properties were revealed; thermal conductivity (10%) and zeta potential (13%) in the presence of 0.8 wt.% of graphite-Al_2_O_3_.	[121]
Graphene	-	Graphene with a concentration of 17.5 mL was reduced polymer usage up to 40% for mud cake formula. Better-quality RP of 13.5lb/gal HPHT was achieved in WBDF without affecting PV and YP.	[122]
Graphitized nanotubes	Homogenization	Decrease of PV with an increase in temperature from 25–85 °C. Value-added RP with an increase of temperature were presented.	[123]
Nano-graphite nanoparticles	Water-in-oil (w/o) micro emulsions	Decrease in FL and RP were enhanced for WBDF.	[124]
Graphene/CNT	Chemical method	Reduced mud filtrate volume up to 18%. Addition of CNT reduced FL, enhanced shale formation. Addition of graphene was decreased friction coefficient from 38–59%. Better lubricity was produced by CNT as compared to graphene at elevated temperature.	[125]
Graphene–SiO_2_	-	Concentration of nanoparticles (0.75 wt.%) yielded better performance in both LPLT and HPHT filtration tests with a reduction of 20.93% and 27.21%, respectively, as compared to the base fluid.	[126]
GO-phosphorylated from welding waste	Chemical synthesis	Addition of GO was tested for improved RP such as PV was reduced from 10 to 7 cp, YP was increased from 11–15 lbs/100 ft^2^, decreased filtrate volume (6 to 3.6 mL) and reduced MCT (1.06 to 0.33 mm), with enhanced lubricity were presented.	[127]

Table 2 includes the following designations; PV: plastic viscosity, SR: shear rate, RP: rheological properties, YP: yield point, ST: shear test, GS: gel strength, FL: fluid loss, MCT: mud cake thickness, CNT: carbon nanotubes, GNP: graphene nanoplates.

### 4.2. Graphene Oxide on Rheological Properties

**Figure 10:**
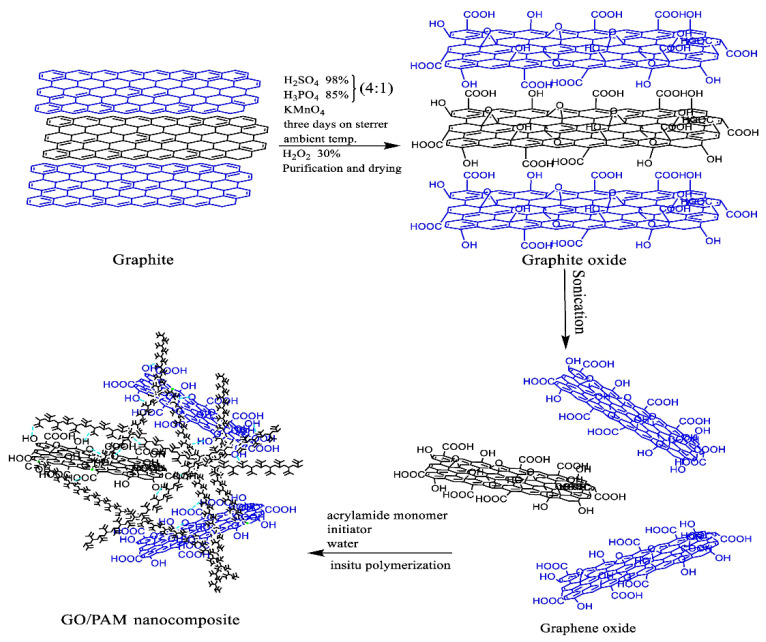
Structural representation of GO/PAM nanocomposite fabrication. Reprinted with permission from [116]. Copyright Elsevier, 2020.

Nanotechnology through the production of nanomaterials has appreciably uplifted a positive impact on the advancement of technology among innumerable industries. Nanomaterials have been found roughly in the size of 1 to 100 nm, provided many advantages upon their applications. In recent years, the effect of GO at HPHT and LPLT conditions have reported high-performance fluid loss control [128]. Upon the discovery of graphene back in 2004 [88], it was proved to be a promising material for many applications. However, due to the difficult top-down synthesis, poor solubility, and agglomeration problem in solution, GO was synthesized from graphite using Hummers method through oxidation [129]. It has retained worthy attributes such as electrical and thermal conductivity, mechanical stiffness and biocompatible properties [130]. Additionally, remarkable mechanical properties were obtained from interfacial interaction of GO with polymer matrix by increasing the functional group of GO sheets to fabricate GO-derived nanocomposites as presented in Figure 10. In this regard, Murphy et al. [131] evaluated important factors, such as plastic viscosity and yield point, through GO as an additive in drilling fluids. They examined rheological behavior as the main factor in adjusting the printability and structure of alginate hydrogels. Consequently, novel properties of GO and their application for modified rheological properties have solved the issues of poor mechanical strength and inadequate structural reliability [132].

Recently, the role of GO in featured rheology was analyzed such as an examination of GO-SiO_2_ nanoparticles for unconventional reservoir shales to reduce cutting dispersion [128], suspension of cutting fluids using GO [133], improved thermal stability and inhibition capabilities of WBDF in Woodford shale [134], the effect of GO functionalization to improve heavy oil recovery [135], clean swelling inhibitor in WBDF [97], and GO as an additive to improve filtration, thermal conductivity, and rheological properties of drilling fluids [116,136].

### 4.3. Graphene Oxide in WBDF

Although, several nanomaterials are considered to maintain the stable rheological and filtration properties of WBDF [137], graphene contains a distinguished thickness of one atom and is known for its evident mechanical, thermal, electrical, and physical properties. However, deprived dispersion of graphene in water has prohibited its usage in WBDF [119].

Newly conducted studies by Kusrini et al. [127] have utilized GO as a superlative alternate and additive in drilling fluids. Production of GO from industrial waste using a modified Hummers method in which graphite goes through chemical oxidation and is followed by its reduction. Due to the highly hydrophilic properties of GO, it is dispersed well in aqueous solution with a wide range of concentrations [92]. From these studies, a comparative analysis of GO was observed to uplift fluid loss performance as compared to the base fluid. The filtrate loss volume was reduced from 6 mL to 3.6 mL and the mud cake thickness was decreased up to 70%. Also, GO produced positive results for the rheological properties of drilling fluid such as plastic viscosity, yield point, and gel strength. Addition of GO minimized the viscosity values. It was analyzed due to the reduction in GO size which was observed as nano-sized particles that triggered less friction and minimized the resistance of flow in drilling fluids. Low plastic viscosity values are desired to prevent high-pressure drops which result in the low circulation of fluid [122]. In contrast, yield point and gel strength are increased with the application of GO in drilling fluids. The increase of yield point was examined due to the twigging of particles together and, hence, to overcome the surface energy resulting from increased GO surface area. Furthermore, it improved the ability of the drilling fluid to carry the drill cuttings to the surface. The measured values of gel strength increased due to the attraction forces among well-dispersed GO with other particles presented in the drilling fluid system. An increase in gel strength is desirable, as it helps the drilling fluid system create a gel structure quicker than the base fluid [112]. Lastly, Alkinani et al. [138] directed an equivalent circulation density (ECD) simulation for the application of GO in drilling fluids. The ECD values of GO did not report a dominant difference with the base fluid. The ECD values indicated GO as more suitable for low-pressure wells to reduce the potential risk of circulation loss.

## 5. Limitations and Challenges

**Figure 11:**
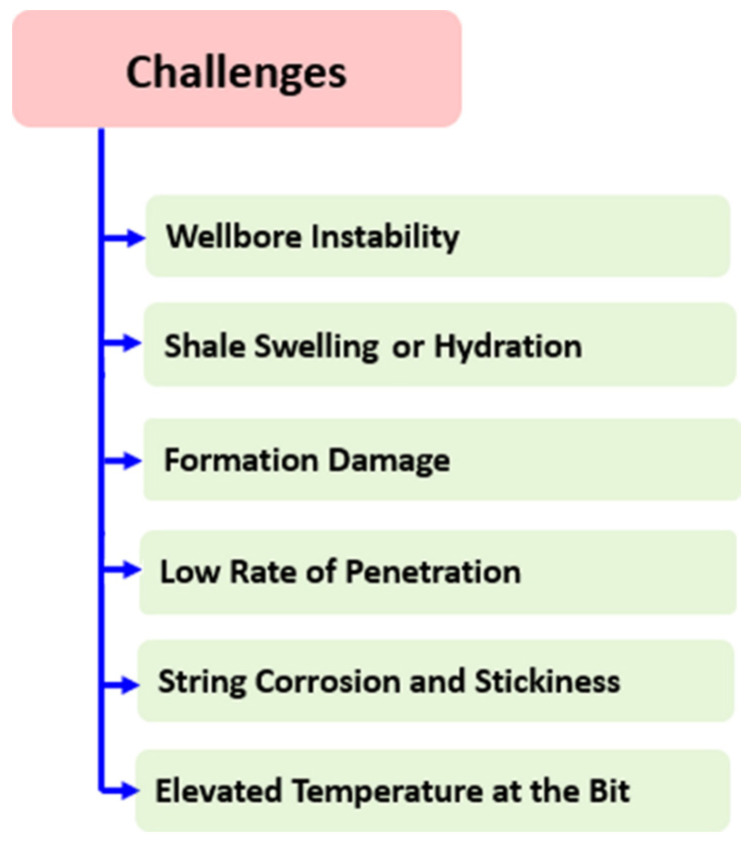
Challenges of drilling operations. Reprinted with permission from [139]. Copyright Elsevier, 2019.

This review endorsed us to report several advantages of nanocomposites as additives for augmenting rheological performance and stable fluid loss control in WBDF. However, a few challenges should be addressed before they can be employed in drilling operations. Several rheological behaviors are uncovered through nanocomposites, for instance, wellbore strengthening, improved shale stability, and drill bit issues as displayed in Figure 11.

The stability of nanocomposite dispersions reports a methodical challenge to maintain drilling fluids. A coherent method for an effective dispersion of nanomaterials in a liquid or solid medium is a crucial phase. In earlier studies, high-speed mixers, magnetic stirrers, ultrasonic baths, ball milling, and other homogenizers are presented [140]. The nano-size of composite mixtures tend to re-aggregate due to the presence of van der Waals forces and confine their high surface area control [141]. Therefore, additives, such as surfactants, stabilizers or ionic liquids, are required to increase the steric hindrance between nanocomposites fabrication and stable dispersions. A brief overview of the advantages and limitations of nanocomposites based on their processing and nanofiller content is presented in Table 3 [142].

Moreover, primary factors, including particle size and morphology, and other structural properties for nanocomposite dispersions are equally important. Researchers have identified challenges during quantitative studies of nanocomposites by addition of suitable additives for the chemical stability of fabricated nanocomposites in WBDF [144]. A comparative analysis of utilizing 0.3 ppb of nanosilica and graphene nanoplates established the ideal effects of graphene nanoplates on filtrate loss in WBDF [91]. It is equally challenging to find an optimized ratio of these nanocomposites for eco-friendly usage. For example, nanocomposites ratios with low concentrations of 0.5 wt.% have influenced the rheological behavior of drilling fluids [145].

Although many types of nanocomposites are commercially available, the cost of their synthesis is still an obstacle for targeted operations in oil and gas industry [146]. The American Petroleum Institute (API) has given particular specifications and procedures which provide difficulty for newly made-up nanocomposites and base fluid formulations due to the fact of their different properties, process parameters, and other requirements [147]. It is also recognized that preparation protocols and key measurements of these nanocomposites find difficulty in the combination with graphene-based materials [148]. In this regard, more research emphasis on graphene-derived materials could be a promising substitute. Significant factors such as mixing time, functionalization of additives, and order of materials doping are crucial and can knowingly affect the drilling fluids behavior. Vital procedures, consistent results, and the role of these nanocomposites in unconventional rheological studies are alarming for researchers and oil companies.

## 6. Future Prospects

An improved description of drilling fluids performance has advanced fundamental aspects in applied rheology. Therefore, potential analysis and extensive methodologies are essential for future research. More studies should endeavor for combinations of novel nanocomposites with graphene-based materials such as graphene-doped nano-additives and GO synthesized through novel analytical techniques. Moreover, a comprehensive quantitative analysis of these nanocomposites should be performed for drilling fluid operations. Further research should be focused on key mechanisms of interaction between nanocomposites and other additives available in drilling fluids. Apart from WBDF, optimizations of fluid formations can possibly be done by adding nanocomposites and their comparison with SBDF and OBDF. Deep analysis of these nanocomposites, for instance, bentonite or barite particles, should be compared to conventional base fluids at elevated pressure and temperature conditions. To reduce formation damages of mud cakes, advanced procedures should be utilized for the characterization and quantification of mud cakes. Several studies have entailed least exposed rheology through nanocomposites, i.e., plastic viscosity, shear rate, yield point, and filtrate loss. It is motivational for upcoming practices to take an account of complete rheograms for filtration tests. Incorporation of such nanocomposites shows a need to combat high pressure and temperature conditions encountering low shear rates and complicated information regarding cuttings, wellbore strengthening, and advancement of these techniques in several types of drilling fluids. Hereafter, the role of graphene-derived nanocomposites will have a major role in the preparation of novel additives for WBDF. It will advance a breakthrough in augmenting the efficiency of drilling operations and expand the overall competitiveness of industrial applications.

## 7. Conclusions

In summary, we demonstrate the usage of nanocomposites in drilling operations with their considerably amplified performance and functionality. It was shown that applications of nanocomposites by a combination of two or more nanomaterials were embedded in a matrix phase. However, challenges are required to resolve for advance production of nanocomposites. By comparing several types of nanocomposites behavior, it was revealed that graphene-derived nanocomposites, particularly, GO-nanocomposites, as additives enhanced the rheological properties of WBDF. This reflects the examination of key factors in producing nanocomposites, such as nanoparticles or other nanomaterials, combined with graphene, and examination of other rheological properties under extreme conditions. However, they have been observed to be expensive and found to be produced in small amounts. Promising attempts were displayed for the modification of plastic viscosity, yield point, gel strength, and filtrate loss at LPLH and HPHT by using graphene-derived nanocomposites. This leads the drilling industry to focus on the commercial production of nanocomposites, either through green synthesis or beyond the laboratories and consolidation of these materials into the end product preserving their nanostructures. Handling of these nanomaterials also paves the way to study a major role of these nanocomposites for OBDF as driving factors. Therefore, novel methods are needed to produce grapheneaceous nanocomposites on a large scale and at an affordable cost, prior to the applications of nanocomposites in WBDF. To conclude, this review will be helpful for researchers to discover novel routes of nanocomposites synthesis, their fabrication with graphene-grafted innovative nanomaterials, and their utilization in several unindicated rheological properties of drilling fluids. The addition of these methods could be equally helpful for future perspectives of modest comparison for a critical variety of drilling fluids.

## Figures and Tables

**Table 1:** Summary of various nanocomposites in rheological properties of WBDF.

Nanocomposites	Experimental Conditions	Rheological Properties	Outcomes	References
Nanosilica polymer composite	LPLT and high temperature up to 446 °F	PV, YP, GS, FL, MCT	Under LPLT conditions, the usage of nanosilica (1.0 wt.%) has greatly enhanced the rheological and filtration properties. Nanocomposites have shown no decomposition under high temperatures at 392, 410, 428, and 446 °F, proving nanocomposites to be suitable under HPHT conditions.	[17]
ZnO nanocomposite	HPHT at 109 to 370 °F and 150 to 18,500 psi	PV, YP	2.3 wt.% of ZnO nanocomposite (5 to 50 nm) resulted in upgrading the rheological properties under HPHT conditions.	[27]
TiO_2_-polyacrylamide	LPLT	PV, YP, GS, FL	Development in rheological properties and filtration behavior under LPLT condition was observed by using 1–14 g of TiO_2_-PAM nanocomposite.	[26,48]
ZnO-polyacrylamide	At 80 and 150 °F	PV, AV, YP, GS, FL	By adding 0.8 g of ZnO-PAM nanocomposite in the drilling fluid, PV and YP increased by 18.8% and 16.7%, respectively. Fluid loss was reduced by 12.7% and 23% under LPLT and HPHT conditions, respectively, when using 1 g of nanocomposite.	[28]
Sepiolite	LPLT and HPHT at 122 to 356 °F and 500 to 6000 psi	-	The experiment showed that WBDF samples with 1.4 wt.% of sepiolite enriched the rheological properties at 6000 psi and temperatures up to 356 °F conditions.	[49]
Polyacrylamide-grafted polyethylene glycol nanosilica	High temperature up to 203 °F	PV, YP, GS, FL	Enhancement of rheological and filtration properties was observed with 0.7 wt.% of nanocomposite and the values remained stable under a temperature of 203 °F.	[50]
Hydrophobic modified polymer-based silica	LPLT	PV, YP, GS, FL	Rheological and filtration properties were improved by adding 2.0 wt.% before and after hot rolling under 250 °F for 16 h.	[51]
Polyethylene glycol grafted nanosilica	LPLT	PV, AV, YP, GS, FL	Results have showed that PV, YP, and AV values were increased while fluid loss volume was decreased to 15.2% by adding 1 g of nanocomposite in drilling fluid.	[52]
Amphiphilic polymer/nano-silica	LPLT	PV, AV, YP, GS, FL	PV was enhanced by the addition of 7.1% nanocomposite. Addition of 2 wt.% nanocomposite reduced the fluid loss volume to 6.4 mL.	[53]
Nanocarboxylmethyl cellulose/polystyrene core-shell nanocomposite	LPLT	PV, AV, YP, GS, FL	PV and AV increased by up surging the concentration of three additives. YP values were the highest for bulk CMC while core-shell nanocomposites recorded the lowest amount of fluid loss volume.	[24]
CuO/ZnO/synthetic polymer nanocomposite	LPLT and high temperature up to 400 °F	PV, YP, GS, FL, MCT	The drilling fluid exhibited stable rheological and filtration properties at 400 °F. Under LPLT conditions, low fluid loss volume was recorded. while the mud cake formed was thin and impermeable.	[54]
Lignosulfonate/Acrylamide graft copolymers	78 °F and 250 °F	PV, YP, GS, FL, MCT	At a temperature of 78 °F and 250 °F, rheological and filtration properties of the drilling fluid were enhanced with the inclusion of nanocomposite (2.4–3.5 g/350 mL water).	[55]
Hybrid polymer nanocomposite poly(styrene-methylmethacrylate-acrylic acid)/nanoclay	High temperature up to 250 °F	PV, YP, GS, FL	The nanocomposite presented stable rheology at temperatures up to 250 °F, and the combination of nanocomposite in nanoclay-based drilling fluid was reduced by up to 22% fluid loss under LPLT conditions, and a 65% reduction in the polymer-based drilling fluid.	[56]
Novel synthetic based acrylamide-styrene copolymer	High temperature up to 250 °F	PV, YP, GS, FL, MCT	Rheological and filtration properties proved a progressive fluid loss control. An ideal filtration performance at LPLT and HPHT conditions was achieved with the addition of 3 g of nanocomposite into the drilling fluid.	[57,58]

Table 1 includes the following designations: WBDF: water-based drilling fluids, LPLT: low pressure-low temperature, HPHT: high pressure-high temperature, PAM: polyacrylamide, CMC: carboxymethyl cellulose, PV: plastic viscosity, YP: yield point, GS: gel strength, FL: fluid loss, MCT: mud cake thickness.

**Table 3:** Advantages and disadvantages of nanocomposites synthesis routes [143].

Synthesis Routes	Nanofiller Content	Advantages	Limitations
In situ polymerization	5–70 wt.%	Fabrication and polymerization occur at the same time to produce an efficient interface between filler and polymer	Suitable for limited types of polymers
Shear press	60–70 wt.%	Fine alignment	Restricted to small-scale production
Vacuum-assisted polymer infiltration	5–70 wt.%	Competent at producing large and complex composites	Filler fractions and thickness are challenging to control
Spray winding	50–80 wt.%	Satisfactory alignment and large-scale production	Comparatively complex apparatus
Capillary rise infiltration	40–60 wt.%	User-friendly apparatus	Limited to thermoplastic polymers
